# Stages of change of the readiness to quit smoking among a random sample of minority Arab -male smokers in Israel

**DOI:** 10.1186/s12889-015-1950-8

**Published:** 2015-07-16

**Authors:** Nihaya Daoud, Samah Hayek, Ahmad Sheikh Muhammad, Kathleen Abu-Saad, Amira Osman, James F. Thrasher, Ofra Kalter-Leibovici

**Affiliations:** Department of Public Health, Faculty of Health Sciences, Ben-Gurion University of the Negev, P.O. Box 653, Beer Sheva, 84015 Israel; School of Public Health. University of Haifa, Haifa, Israel; Cardiovascular Epidemiology Unit, Gertner Institute for Epidemiology and Health Policy Research, Sheba Medical Center, Ramat Gan, 52621 Israel; Department of Health Promotion, Education, and Behavior, Arnold School of Public Health, University of South Carolina, 800 Sumter Street, Columbia, SC 29208 USA; Sackler Faculty of Medicine, Tel-Aviv University, Tel-Aviv, Israel

## Abstract

**Background:**

Despite advanced smoking prevention and cessation policies in many countries, the prevalence of cigarette smoking among indigenous and some ethnic minorities continues to be high. This study examined the stages of change (SOC) of the readiness to quit smoking among Arab men in Israel shortly after new regulations of free-of-charge smoking cessation workshops and subsidized medications were introduced through primary health care clinics.

**Methods:**

We conducted a countrywide study in Israel between 2012–2013. Participants, 735 current smokers; 18–64 years old; were recruited from a stratified random sample and interviewed face-to-face using a structured questionnaire in Arabic. We used ordered regression to examine the contribution of socio-economic position (SEP), health status, psychosocial attributes, smoking-related factors, and physician advice to the SOC of the readiness to quit smoking (pre-contemplation, contemplation and preparation).

**Results:**

Of the current smokers, 61.8 % were at the pre-contemplation stage, 23.8 % were at the contemplation stage, and only 14.4 % were at the preparation stage. In the multinomial analysis, factors significantly (P < 0.05) contributing to contemplation stage compared to pre-contemplation stage included [odds ratio (OR), 95 % confidence interval (CI)]: chronic morbidity [0.52, (0.31-0.88)], social support [1.35, (1.07-1.70)], duration of smoking for 11–21 years [1.94, (1.07-3.50)], three or more previous attempts to quit [2.27, (1.26-4.01)], knowledge about smoking hazards [1.75, (1.29-2.35)], positive attitudes toward smoking prevention [1.44, (1.14-1.82)], and physician advice to quit smoking [1.88, (1.19-2.97)]. The factors significantly (P < 0.05) contributing to preparation stage compared to pre-contemplation stage were [OR, (95 % CI)]: chronic morbidity [0.36, (0.20-0.67)], anxiety [1.07, (1.01-1.13)], social support [1.34, (1.01-1.78)], duration of smoking 5 years or less [2.93, (1.14-7.52)], three or more previous attempts to quit [3.16, (1.60-6.26)], knowledge about smoking hazards [1.57, (1.10-2.21)], and positive attitudes toward smoking prevention [1.34, (1.00-1.82)].

**Conclusions:**

Most Arab men who currently smoke are in the pre-contemplation stage, indicating low readiness to quit smoking. New policies of free-of-charge smoking-cessation group sessions and subsidized medications introduced through primary health care clinics in Israel may be less effective among Arab men. For these policies to promote cessation more successfully, tailored interventions and campaigns may be needed to increase the readiness to quit smoking in this population, especially for those at the pre-contemplation stage.

## Background

Smoking remains a major global public health concern despite its decreasing prevalence in developed counties [1, 2]. Smoking is the leading preventable cause of chronic morbidity and mortality [3]. On average, the life expectancy of smokers is reduced by almost 10 years [4]. Smoking cessation can decrease the risk of smoking-related diseases and mortality. Smokers who stop smoking before the age of 35 can enjoy the same life expectancy as non-smokers [3]. Smokers who quit at the age of 60, 50, 40 and 30 can increase their life expectancy by 3, 6, 9 and 10 years, respectively [4]. For those who quit before the age of 50, the risk of myocardial infarction can be reduced by half one year after smoking cessation [5, 6]. Smoking cessation can also reduce the cost of treating smoking-related diseases [6]. However, although many smokers express interest in quitting, only 2-5 % succeed annually [7].

Behavioral and pharmacological interventions have been shown to be effective in smoking cessation [8]. The stages of change (SOC) theory has been used in many behavioral interventions to facilitate smoking cessation [9, 10]. The SOC assumes that smoking cessation is a process of movement through five motivational stages [11, 12], each representing a different temporal and motivational aspect of behavioral change [13]. The first three out of five stages describe individuals’ readiness to quit smoking. These stages include: a. pre-contemplation, when smokers have no intention to quit; b. contemplation, when smokers express an intention to quit smoking within the next 6 months; and c. preparation, when smokers plan to quit smoking within the next 30 days. When individuals quit smoking for 6 months, they are at the action stage. They are considered to be at the maintenance stage if they have been abstinent for 6 months to 5 years, and at the termination stage if they have been abstinent for more than 5 years. A study by DiClemente and colleagues found that stage differences predict attempts to quit smoking and cessation success at 1- and 6-month follow-up [13].

Interventions tailored to an individual’s readiness to quit smoking based on SOC can be more effective in facilitating quitting success [14–17]. Prochaska et al. [18]. suggested employing different strategies called “process of change” to promote behavioral change across different SOC. Rosen et al. [19]. conducted a meta-analysis of studies evaluating the process of change and found that cognitive processes such as “consciousness raising” were more effective among smokers at the pre-contemplation and contemplation stages. Assessing the SOC of the readiness to quit smoking among smokers in health care service settings can help in planning more effective interventions for cessation [11, 20]. However, smoking cessation policies and interventions do not always consider the SOC of the readiness to quit in program planning. This is particularly important when there are different population groups who might be at different stages in their readiness to quit smoking.

The World Health Organization (WHO) policy report on smoking cessation shows that many countries implement the MPOWER plan, which includes monitoring tobacco use, implementing prevention policies, protecting people from tobacco smoke, offering help to quit tobacco use, warning about the dangers of tobacco, enforcing bans on tobacco advertising, promotion and sponsorship, promoting policies to quit smoking, and raising taxes on tobacco [21]. However, smoking cessation is a neglected area in the initial period of the Framework Convention on Tobacco Control (FCTC) adoption and implementation [22]. Despite the availability of nationwide tobacco control programs and policies in many countries [23], smoking cessation remains a challenge, particularly among some ethnic minorities [24–27]. For example, the prevalence of smoking is higher among Aborigine peoples in Australia [28] and Canada [29], racial and ethnic minorities in the UK [30], and some ethnic minorities in the US [31], compared to the majority population groups. Generally, few studies have been conducted on smoking cessation in ethnic minority populations [32]. Thus, it remains unclear as to why smoking cessation programs are less effective among minority groups [25, 26]. A study in a health care setting in the US showed that Black and Hispanic individuals were less likely to receive and use smoking cessation interventions [33]. Researchers argue that universal policies for smoking cessation can increase inequalities in smoking since such policies are less effective in minority groups [34]. Therefore, smoking cessation interventions need to be tailored to different population groups and include assessment of the SOC of the readiness to quit at baseline [27, 35]. This requires more research on disadvantaged and marginalized subgroups of society [32].

In Israel, despite implementation of the WHO MPOWER plan of tobacco control and multiple national programs and interventions, there are persistent inequalities in rates of smoking between population groups. Of special concern is the very high prevalence of smoking among Arab minority men [36]. In all age groups, smoking prevalence is 1.5-2.2 times higher among Arab men compared to Jewish men [36]. In 2011, smoking prevalence was 43.8 % among Arab men compared to 23.7 % among their Jewish counterparts [36]. In addition to higher prevalence of smoking, Arab men smoke more frequently than Jewish men. According to the Israeli Ministry of Health report (2011), among smokers, 30.9 % of Arab men smoke more than one cigarette pack a day compared to only 12.6 % of Jewish men [36]. Smoking has been attributed to increased incidence of lung cancer among Arab men [37]. However, little is known about the SOC of the readiness to quit smoking among Arab men in Israel.

Despite a higher prevalence of smoking, few studies have been conducted on smoking cessation and intention to quit among individuals in Arab countries. One study found high rates of smoking in Middle Eastern countries, ranging from 30 % to 60 % [38]. Between 1990 and 2009, cigarette consumption increased by 57 % in the Middle East and Africa but dropped by 26 % in Western Europe [39]. High prevalence of smoking in the Mediterranean region had implications for smokers’ readiness to quit and might translate into a higher proportion of smokers being at the pre-contemplation stage.

In the current study, we applied the SOC theory to assess the readiness to quit smoking and examined factors that might contribute to pre-contemplation, contemplation, and preparation in a random sample of Arab minority men in Israel. The study was conducted shortly after new regulations of free-of-charge workshops and subsidized medications were introduced in primary health care clinics in 2010. Therefore, the current study might provide baseline information needed to assess whether the new health policies of free-of-charge workshops and subsidized medications are effective in changing the SOC of the readiness to quit among Arab male smokers.

## Methods

### Sampling

We conducted a cross-sectional study between 2012 and 2013. The study included a nationwide stratified random sample of Arab men. Individuals were eligible if they were between 18 and 64 years of age, tobacco smokers, Arab citizens in Israel, and living in an Arab town of 5,000 or more inhabitants. The study participants were randomly selected from the Israeli population registry. The sample design took into account the Arab population distribution according to area of residence, locality size and socio-economic status. Sixty-four Arab towns with 5,000 or more residents were identified. For the purpose of creating the sample strata, the country was divided into three major regions in Israel: North, Center and South, containing 58 %, 30 % and 12 % of the Arab population, respectively. Within each region, towns were stratified according to population size (5,000-9,999, 10,000-29,999, and 30,000 or more residents) and three ranks of socio-economic score of the locality according to the Central Bureau of Statistics. For each region, one town was sampled from each size and socio-economic specific stratum. The final sample consisted of 20 towns. The number of persons sampled and interviewed in each locality was proportional to the size of the entire population of the respective stratum. Information that identifies the men sampled from the population registry was received (first name, last name, ID and address) from the Ministry of Interior (approximately 15,000 men). We prepared two random samples of Arab men. The size of each sample was at least three times the number of interviews for replacement of participants, to account for lack of identification or refusal to interview.

We used STATA software (version 13) for power calculation and sample size. This was based on several assumptions: 1. The prevalence of Arab men smokers at the pre-contemplation stage will be 50 % based on previous reports in this population [36] and on results about smokers at the pre-contemplation stage among Arab immigrants in Australia; [40] 2. Based on the findings by Wewer et al., [41] there will be approximately a 10 % difference in the proportion of individuals at the pre-contemplation stage between the 18–24 and 45–64 age groups (58.4 % and 68.0 %, respectively). The needed sample size is 964 at a significance level α of 5 % and 80 % power. This sample size also took into consideration 0.005 for Inter Cluster Correlation within towns, as this is a stratified sample [42].

### Data collection

This study was approved by the Ethics Review Board of the Faculty of Health Sciences at Ben-Gurion University of the Negev. Data were collected between September 2012 and September 2013. Men were contacted via telephone calls or home visits and screened for their tobacco smoking status. Men who met the eligibility criteria and agreed to participate in the study signed an informed consent form and were interviewed face-to-face in their home by trained Arab-speaking interviewers using a structured questionnaire in Arabic. Out of 1,620 men who were approached, 1,165 men met the eligibility criteria (current or past smokers) and 964 agreed to participate (response rate = 83 %). Of those, 735 were current smokers and formed the study population for this paper.

### Measures

#### The stages of change (SOC) of the readiness to quit smoking

Was measured using the following questions: “Do you smoke?” Participants who replied positively were coded as current smokers. They were then asked if they intend to quit smoking. Response categories were “no, never” (pre-contemplation stage), “yes, thinking to quit within the next 6 month” (contemplation stage), and “yes, thinking to quit within the next month” (preparation stage).

***Sociodemographic characteristics*** included age groups according to quintiles (18–25, 26–35, 36–45 and 46–64 years of age), and marital status (married or unmarried).

***Socioeconomic position*** (*SEP*) was determined by three variables:

*Education level*: The highest educational institution the participant completed (“less than high school,” “high school” and “more than high school”).

*Primary source of income*: The main source of household income (from “work” or “social allowance and other”).

*Employment status*: The participant’s current employment status (“yes, full time employment” and “part-time work or unemployed”).

#### Health status included two variables

*Chronic disease*: A yes / no question; if the participant was ever informed by a physician that he has a chronic disease.

*Anxiety level* was measured by the “State-Trait Anxiety Inventory” (STAI) [43, 44], a commonly used measure [45, 46]. We used ten items from the original scale that assesses trait and state anxiety. Examples of state anxiety items include: “I am tense”, “I am worried”, “I feel calm”, and “I feel secure”. Examples of trait anxiety items include: “I worry too much over something that really doesn’t matter”, “I am content”, and “I am a steady person”. Response categories include: 1. almost never, 2.sometimes, 3.often, and 4.almost always. The codes of positively worded items were reversed. The ten items were summed to create a total score of anxiety (range 1–40). Higher scores indicate greater anxiety levels. Cronbach’s alpha of the scale was 0.796, indicating high internal consistency.

#### Psychological factors included two variables

*Stressful life events* measures exposure to nine stressful events during the previous year (e.g., unemployment, major family problems) [47]. The list includes a modified version of the recent life events index [48]. Respondents were asked: “For each situation, indicate whether it have happened to you or to close family members (i.e., spouse, children, parents, or close friends) in the last year: Did you or someone in your family 1) Get fired from work? 2) Had a major financial crisis? 3) Experienced death of a family member or a close relative? 4) Get sick or injured? 5) Experienced a change of job to lower skill one? 6) Begin receiving income support or go on welfare? and 7) Experienced any other difficult life events in the last year?” The modified questions include: “During the past four years: 8) Did you or someone in your family get injured in events related to the Arab-Israeli conflict (e.g., a terror attack, Nakba day, protests)? and 9) Did you or someone in your family spend time in prison?” Responses categories were yes\no. We summed the response scores across all items to create a count variable of stressful life events (range 0–9). Higher scores indicate higher levels of exposure.

*Social support* was measured by a scale of six items assessing material, emotional and informational support [49]. The general question was: “To what extent do you have the following types of support?”; Examples for types of support: 1. Someone who provides you with advice during difficult times; 2. Someone who helps you materially, such as takes you to the doctor, etc.; and 3. Someone you trust and talk to about your daily problems. Response categories were: 1. never, 2. rarely, 3. sometimes, 4. frequently, and 5. always. We calculated the mean scores for the answers (range 1–5). Higher scores indicate a higher level of social support. Internal consistency of the scale was high (Cronbach’s alpha = 0.87) in prior research among Arab populations in Israel [50]. In the current study Cronbach’s alpha was 0.91.

#### Smoking-related variables included direct questions regarding

*Age at initiation of smoking* was grouped into three categories: 15 years or less, 16–18 years, and 19 years or more. These categories are closely related to the age quartiles of our study sample.

*Duration of smoking* was classified into four categories: 5 years or less, 6–10 years, 11–20 years, and 21 years or more.

*Number of cigarettes smoked per day*: Current smokers were asked: “on average, how many cigarettes per day do you smoke?” Response categories were “10 cigarettes or less”, “11-20 cigarettes”, “21-30 cigarettes”, and “31 cigarettes or more”.

*Number of previous attempts to quit* was measured by a direct question: “How many times did you try to quit smoking?” Answers were grouped into three categories: never, once or twice, three times or more.

*Nicotine dependence level* was measured by the FrangestÖm Test for Nicotine Dependence (FTND) [51]. This measure includes six items: three yes/no items are scored 0 (no) and 1 (yes) and three multiple-choice items are scored from 0 to 3. The items were summed to yield a total score of 0–10. Classification of dependence is as follows: 0–2 very low, 3–4 low, 5 moderate, 6–7 high, 8–10 very high. In the current study this variable was used as continuous variable, with higher scores indicating greater nicotine dependence.

*Knowledge about smoking hazards* included the sum score of three yes/no questions: “Do you think smoking can harm your ability to do activities that you are used to do day by day?” “Do you think that smoking can harm organs within the body?” and “Do you think smoking can cause diseases?” The answers were summed into one score. This was a continuous variable in the current study, with higher scores indicating greater knowledge (range: 0–3).

*Attitudes towards smoking prevention and cessation* were measured by the mean score of the level of agreement to three statements: “To what extent do you: 1. Support the anti-smoking law in the public places? 2. Think that there is a need to enforce smoking prevention and treatment in the Arab society in Israel? 3. Think that smoking is a common habit among Arab men?” Response categories were: “strongly agree,” “agree,” “don’t agree,” and “don’t agree at all”. This was a continuous variable in the current study, with higher mean scores indicating greater levels of agreement (range 0–3).

*Smoking social environment* included the number of smokers in the participant’s home in addition to himself (none, one, and two or more), parental smoking (yes/no), and indoor smoking (yes/no).

*Physician advice to quit*: An answer to a yes/no question assessing if the participant had ever been advised by his family physician to quit smoking.

### Data analysis

Data analysis was conducted using SAS (Version 9.3. Cary, NC: SAS Institute Inc. 2011). We examined the univariate association between the independent variables and the SOC of the readiness to quit smoking (pre-contemplation, contemplation and preparation), using Chi-square test for categorical variables, and Kruskel-Wallis for the independent continuous variables. Independent variables that were significantly (p < 0.05) associated with the SOC of the readiness to quit smoking in the univariate analysis were included in ordered logistic regression analysis after the proportional odds assumption was tested. Pre-contemplation was the reference category for the other two categories (contemplation and preparation).

## Results

**Fig. 1:**
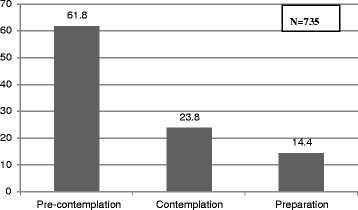
Stages of change of the readiness to quit smoking among Arab men smokers in Israel

The SOC of the readiness to quit smoking are presented in Fig. 1. Of the sample, 61.8 % were at the pre-contemplation stage, 23.8 % were at the contemplation and 14.4 % were at the preparation stage.

The distribution of the study variables is presented in Table 1. The average age of the participants was 36.42 years (SD = 12.88) and 63.4 % were married. Most participants (83.2 %) had high school education or less. The primary source of income for 16.1 % of the participants was social allowance, and 26.7 % were unemployed or had a part time job. About 1 in 3 (30.9 %) men reported having a chronic disease. The average score for anxiety was moderate (mean = 16.03, SD = 4.35) and the average score for stressful events was low (mean = 0.79, SD = 1.29). The mean social support score was moderate (mean = 3.6, SD = 0.97).

**Table 1:** Socio-demographic characteristics of current smokers (N = 735)

		Total sample
	N	%
Age		
18-25	204	27.83
26-35	178	24.28
36-45	149	20.33
46-64	202	27.56
Marital status		
Unmarried	269	36.65
Married	465	63.35
*Socio-economic position*		
Education level		
Less than high school	163	22.39
High school	443	60.85
More than high school	122	16.76
Primary source of income		
Social allowances/other	113	16.14
Work	587	83.86
Employment status		
Unemployed or part time	195	26.71
Full time employment	535	73.29
*Health status*		
Chronic disease		
Yes	227	30.88
No	508	69.12
Anxiety^1^ (mean (SD) range)	733	16.03 (4.35) 5-39
*Psychosocial factors*		
Stressful life events^1^ (mean (SD) range)	735	0.79 (1.29) 0-9
Social support^1^ (mean (SD) range)	735	3.66 (0.97) 1-5
*Smoking-related variables*		
Age at smoking initiation		
≤15 years old	133	18.29
16-18 years	365	50.21
≥19 years old	229	31.50
Duration of smoking		
≥21 years	238	36.56
11-20 years	155	23.81
6-10 years	133	20.43
5 years or less	125	19.20
Number of cigarettes per day		
≥31 cigarettes	75	11.42
21-30 cigarettes	171	26.03
11-20 cigarettes	260	39.73
≤10 cigarettes	150	22.83
Number of previous attempts to quit		
Never	434	60.78
Once or twice	158	22.13
Three times or more	122	17.09
Nicotine dependence score (FTND)^1^ (mean (SD) and range)	672	4.17 (2.61) 0-10
Parents smoking		
Yes	396	45.30
No	328	54.70
Number of smokers at home		
Two or more	285	39.80
One	230	32.12
None	201	28.07
Smokes indoors		
Yes	492	67.58
No	236	32.42
Knowledge about the smoking hazards^1^ (mean (SD) range)	735	2.45 (0.89) 0-3
Attitudes towards smoking prevention and cessation^1^ (mean (SD) range)	735	2.19 (1.03) 0-3
Physician advice to quit smoking		
No	444	60.57
Yes	289	39.43

^1^For continuous variables, mean, standard deviation and range are presented

Half of the participants (50.2 %) began smoking between 16 and 18 years of age. Long-term smoking was evident: 36.6 % had been smoking for more than 21 years. About 37.5 % smoked 21 cigarettes or more per day. Three fifths (60.8 %) never attempted to quit smoking. The nicotine dependence score was moderate (mean = 4.17, SD = 2.61). The prevalence of at least one parent who smoked was high (45.3 %). Over two-thirds (71.9 %) stated that they live with one or more smokers at home and 67.6 % reported indoor smoking. The participants had a high mean score on the knowledge about smoking hazards (mean = 2.45, SD = 0.89), and a high mean score on positive attitudes towards smoking prevention (mean = 2.19, SD = 1.03). More than half (60.6 %) stated that their primary care physician did not advise them to quit smoking.

Results of the bivariate analysis are presented in Table 2. Marital status, education level, presence of a chronic disease, anxiety, and social support were significantly associated with the SOC of the readiness to quit smoking. A high proportion of the participants at the pre-contemplation stage (Table 2) were unmarried (65.8 %) (P = 0.039, *X*^2^ = 6.46, df = 2) and had a high school education (63.7 %) (P = 0.023, *X*^2^ = 11.3, df = 4). In addition, they had medium anxiety levels (mean = 15.9, SD = 4.4) (p < 0.001, *KW* = 14.17, df = 2) and a medium social support score (7.04 %) (P = 0.047, *KW* = 1.63, df = 2). They were also less likely to have chronic disease(s) (66.3 %) (P < 0.001, *X*^2^ = 14.56, df = 2). Age, primary source of income, employment status, and stressful life events were not significantly associated with the SOC of the readiness to quit smoking.

**Table 2:** Bivariate associations: stages of change to quit smoking by socio-demographic characteristics, socio-economic position, health status and psychological wellbeing (N = 735)

	Pre-contemplation		Contemplation		Preparation		P-value*
	N = 454		N = 175		N = 106		
	n	%	n	%	n	%	
Age							0.058
18-25	136	66.67	32	15.69	36	17.65	
26-35	107	60.11	47	26.40	24	13.48	*X* ^*2*^ *= 12.18*
36-45	93	62.42	37	24.83	19	12.75	*DF = 6*
46-64	116	57.42	59	29.20	27	13.37	
Marital status							0.039
Unmarried	177	65.80	50	18.60	42	15.60	*X* ^*2*^ *= 6.46*
Married	276	59.36	125	26.88	64	13.76	*DF = 2*
*Socio-economic position*							
Education level							0.023
Less than high school	101	61.97	44	26.99	18	11.04	*X* ^*2*^ *= 11.33*
High school	282	63.66	102	23.02	59	13.32	*DF = 4*
More than high school	66	54.10	27	22.13	29	23.77	
Primary source of income							0.263
Social allowance/ other	77	68.14	25	22.12	11	9.74	*X* ^*2*^ *= 2.65*
Work	360	61.33	139	23.68	88	14.99	*DF = 2*
Employment status							0.545
Unemployed or part time	114	58.46	49	25.13	32	16.41	*X* ^*2*^ *= 1.20*
Full time employment	335	62.62	126	23.55	74	13.83	*DF = 2*
*Health status*							
Chronic disease							
No	337	66.34	1076	29.96	64	18.50	<0.001
Yes	117	51.54	8	21.06	42	12.60	*X* ^*2*^ *= 14.56*
							*DF = 2*
Anxiety^a^	453	15.92 (4.40)	175	15.56 (4.21)	105	17.25 (4.21)	<0.001^b^
							*KW = 14.17*
							*DF = 2*
*Psychosocial factors*							
Stressful life events^a^	454	0.76 (1.21)	175	0.81 (1.45)	106	0.88 (1.23)	0.440^b^
							*KW = 1.63*
							*DF = 2*
Social support ^a^	454	7.04 (4.35)	174	6.65 (4.62)	106	7.80 (4.15)	0.047^b^
							*KW = 6.11*
							*DF = 2*

*P value indicates the differences in the distribution of the variable of interest across the 3 levels of the stage of change

^a^For continuous variables, mean and standard deviation are presented

^b^Kruskal Wallis (KW) for these associations otherwise *X* ^*2*^ test

We further examined the associations between each of the smoking-related factors and SOC of the readiness to quit. Table 3 shows that duration of smoking, previous attempts to quit smoking, number of smokers at home, smoking indoors, knowledge about smoking hazards, attitudes towards smoking prevention and cessations, and receiving physician advice to quit smoking were significantly associated with the SOC of the readiness to quit smoking. A high proportion of participants at the pre-contemplation stage had long-term (≥21 years; 67.8 %) or short-term (≤5 years; 67.2 %) duration of smoking (P = 0.025, *X*^2^ = 14.5, df = 6), never attempted to quit smoking (71.9 %) (P < 0.001, *X*^2^ = 53.7, df = 4), had two or more smokers at home (66.3 %) (P = 0.032, *X*^2^ = 10.5, df = 4), and had smoked indoors (65.9 %) (P < 0.001, *X*^2^ = 12.6, df = 2). In addition, those at the pre-contemplation stage had low scores on the knowledge about smoking hazards (mean = 2.30, SD = 0.99), held less positive attitudes towards smoking prevention and cessation (mean = 2.01, SD = 1.11), and were less likely to receive physician advice to quit smoking (68.5 %). The age at which individuals began smoking, the number of cigarettes smoked per day, the nicotine dependence scores, and parental smoking were not significantly associated with the SOC.

**Table 3:** Bivariate associations: Stages of change by smoking-related factors (N = 735)

	Pre-contemplation		Contemplation		Preparation		P-value*
	N = 454		N = 175		N = 106		
	n	%	n	%	n	%	
Age at smoking initiation							
≤15 years old	86	64.66	24	18.05	23	17.29	0.367
16-18 years	227	62.19	88	24.11	50	13.70	*X* ^*2*^ *= 4.29*
≥19 years old	136	59.39	62	27.07	31	13.54	*DF = 4*
Cigarettes per day							0.247
≥31 cigarettes	39	52.00	21	28.00	15	20.00	
21-30 cigarettes	113	66.08	36	21.05	22	12.87	*X* ^*2*^ *= 7.87*
11-20 cigarettes	157	60.15	72	27.59	32	12.26	*DF = 6*
≤10 cigarettes	96	64.00	31	20.67	23	15.33	
Duration of smoking							0.025
≥21 years	147	67.76	62	26.05	29	12.18	
11-21 years	85	54.84	42	27.10	28	18.06	*X* ^*2*^ *= 14.51*
6-10 years	88	66.16	31	23.31	14	10.53	*DF = 6*
5 years or less	84	67.20	17	13.60	24	19.20	
Previous attempts to quit							
Never	312	71.89	82	18.89	40	9.22	<0.001
Once or twice	82	51.90	46	29.11	30	18.99	*X* ^*2*^ *= 53.68*
Three times or more	47	38.52	43	35.25	32	26.23	*DF = 4*
Nicotine dependence score (FTND) ^a^	414	4.24 (2.66)	163	4.24 (2.47)	95	3.87 (2.60)	0.421 ^b^
							KW = 1.47
							DF = 2
Parents smoking							
Yes	244	61.62	85	21.46	67	16.91	0.058
No	202	61.59	88	26.83	38	11.58	*X* ^*2*^ *= 5.68*
							*DF = 2*
Number of smokers at home							
Two or more	189	66.32	62	21.75	34	11.93	0.032
One	146	63.48	56	24.35	28	12.17	*X* ^*2*^ *= 10.52*
None	108	53.73	53	26.37	40	19.90	*DF = 4*
Smokes indoors							
No	124	52.54	67	28.39	45	19.07	0.001
Yes	324	65.85	108	21.95	60	12.20	*X* ^*2*^ *= 12.56*
							*DF = 2*
Knowledge about smoking hazards^a^	454	2.30 (0.99)	175	2.66 (0.64)	106	2.71 (0.60)	<0.001^b^
							*KW = 25.35*
							*DF = 2*
Attitudes towards smoking prevention and cessation^a^	454	2.01 (1.11)	175	2.49 (0.81)	106	2.50 (0.60)	<0.001 ^b^
							*KW = 36.94*
							*DF = 2*
Physician advice to quit smoking							<0.001
No	304	68.47	84	18.92	56	12.61	*X* ^*2*^ *= 21.39*
Yes	150	51.90	91	31.49	48	16.61	*DF = 2*

*P- value indicates the differences in the distribution of the variable of interest across the 3 levels of the stage of change

^a^For continuous variables, mean and standard deviation are presented

^b^Kruskel Wallis for these associations otherwise *X* ^*2*^ test

In the ordered multivariable regression results (Table 4), presence of a chronic disease, a higher anxiety score, higher social support score, shorter duration of smoking, higher number of quitting attempts, higher knowledge score about smoking hazards, more positive attitudes towards smoking prevention and cessation, and receiving physician advice to quit smoking were significantly (P < 0.05) associated with the likelihood of being at a more advanced SOC of the readiness to quit smoking. Conversely, number of years of education, marital status, and smoking indoors were not significantly associated with the SOC. The odds in favor of being at the contemplation stage versus the pre-contemplation stage for participants without a chronic disease was half that of participants with a chronic disease (OR = 0.52, 95 % CI = 0.31-0.88). The same directionality of the OR was found when we compared participants in the preparation stage versus the pre-contemplation stage: the odds of not having a chronic disease were a third of having a chronic disease (OR = 0.36, 95 % CI = 0.20-0.67). For a one unit increase in the anxiety score, the odds in favor of being at the contemplation stage versus the pre-contemplation stage were 0.97 (95 % CI = 0.92-1.02), whereas the OR in favor of being in the preparation stage versus the pre-contemplation stage was 1.07 (95 % CI = 1.01-1.13). Higher social support was associated with being in a more advanced stage in the readiness to quit smoking. The OR associated with a one unit increment of social support in favor of being in the contemplation stage versus the pre-contemplation stage was 1.35 (95 % CI = 1.07-1.70), and the OR in favor of preparation stage versus pre-contemplation stage was 1.34 (95 % CI = 1.01-1.78). Participants who had smoked for less than 21 years were more likely to be in a more advanced SOC than long-term smokers. Three or more attempts to quit smoking was the strongest contributing factor to being at a more advanced level in the readiness to quit smoking. The odds in favor of being in the contemplation versus the pre-contemplation stage for those who had three or more attempts to quit smoking were 2.27 (95 % CI = 1.27-4.10) times greater than for those who had never attempted to quit. The same directionality was found among participants in the preparation stage. The odds in favor of being in the preparation versus pre-contemplation stage were 3.16 (95 % CI = 1.60-6.26) times as great as for those who had never attempted to quit smoking in the past. Higher knowledge about smoking hazards was associated with higher SOC of the readiness to quit. For a one unit increase in the knowledge about smoking hazards, the OR of advancement to the contemplation from the pre-contemplation stage was 1.75 (95 % CI = 1.29-2.35). The same directionality was found in the higher SOC. For a one unit increase in the knowledge about smoking hazards, the OR for being in the preparation versus pre-contemplation stage was 1.57 (95 % CI = 1.10-2.21). In addition, more positive attitudes towards smoking prevention and cessation were associated with higher readiness to quit. A one unit increase in positive attitudes toward smoking prevention and cessation increased the OR of being in the contemplation versus pre-contemplation stage by 1.44 (95 % CI = 1.14-1.82). The same directionality was found in the higher SOC. A one unit increase in positive attitudes toward smoking prevention and cessation increased the odds of being in the preparation versus pre-contemplation stage by 1.34 (95 % CI = 1.00-1.82). Receiving physician advice to quit contributed to participants’ advancement from pre-contemplation to contemplation. The OR of being in the contemplation versus pre-contemplation stage among those who received advice from a physician to quit smoking was 1.88 (95 % CI = 1.19-2.97) compared to participants who did not receive such advice. However, receiving physician advice to quit smoking did not increase the likelihood that participants would be in the preparation stage, as this was not a significant association (OR = 1.63, 95 % CI = 0.93-2.77).

**Table 4:** Ordered logistic regression for factors contributing to the stages of change of the readiness to quit smoking (N = 735)

	Pre-contemplation	Contemplation	Preparation	
	N = 454	N = 175	N = 106	P-value
	Reference categories	Odds Ratio (95 % CI)	Odds Ratio (95 % CI)	
Education level				0.356
Less than high school	1	1	1	
High school		0.97 (0.56-1.67)	1.31 (0.63-2.72)	
More than high school		0.81 (0.41-1.64)	1.98 (0.86-4.57)	
Chronic disease				0.002
Yes	1	1	1	
No		0.52 (0.31-0.88)	0.36 (0.20-0.67)	
Anxiety	1	0.97 (0.92-1.02)	1.07 (1.01-1.13)	0.016
Social support	1	1.35 (1.07-1.70)	1.34 (1.01-1.78)	0.017
Marital status				0.623
Unmarried	1	1	1	
Married		0.90 (0.48-1.67)	0.70 (0.34-1.44)	
Duration of smoking				0.025
≥21 years	1	1	1	
11-21 years		1.94 (1.07-3.50)	2.48 (1.18-5.21)	
6-10 years		1.46 (0.71-2.99)	1.35 (0.54-3.40)	
5 years or less		0.96 (0.41-2.27)	2.93 (1.14-7.52)	
Previous attempts to quit				0.007
Never		1	1	
Once or twice	1	1.33 (0.81-2.20)	1.76 (0.95-3.24)	
Three times or more		2.27 (1.26-4.01)	3.16 (1.60-6.26)	
Number of smokers at home				0.210
Two or more	1	1	1	
One		1.41 (0.84-2.38)	1.30 (0.67-2.53)	
None		1.41 (0.80-2.46)	2.06 (1.05-4.04)	
Knowledge about smoking hazards	1	1.75 (1.29-2.35)	1.57 (1.10-2.21)	<0.001
Positive Attitudes towards smoking prevention and cessation	1	1.44 (1.14-1.82)	1.34 (1.00-1.82)	0.004
Smokes indoors				0.309
Yes		1	1	
No	1	1.21 (0.76-1.94)	1.52 (0.88-2.65)	
Physician advice to quit smoking				0.016
No	1	1	1	
Yes		1.88 (1.19-2.97)	1.63 (0.93-2.77)	

## Discussion

The SOC theory has been used extensively to study smoking cessation [9, 35, 52]. However, research on smoking cessation in low-income and marginalized populations is scarce [32]. In particular, few studies have used SOC to examine readiness to quit smoking in Mediterranean countries, where smoking prevalence is increasing or has remained consistently high over time [1, 39, 53]. Our findings regarding the SOC of the readiness to quit smoking among Arab minority men in Israel show that 62 % of the participants were at the pre-contemplation stage, 24 % at the contemplation stage, and 14 % at the preparation stage. These findings indicate lower readiness to quit smoking compared to previous studies in the US and Europe, where 40-45 % of smokers were at the pre-contemplation, 35-40 % at the contemplation, and 20 % at the preparation stage [35]. In addition, our result regarding the percentage of individuals at the preparation stage was lower than what has been reported in Turkey (20 %) [54] and Iran (17 %) [55].

Compared to previous findings on minorities in other countries, our study found a higher proportion of smokers at the pre-contemplation stage. For example, one study of Aboriginal peoples in Canada found that 39.6 % were at the pre-contemplation and 43.4 % were at the contemplation stage [27]. A study among African Americans in the US found that 51 %, 28 % and 17 % were in the pre-contemplation, contemplation and preparation stages, respectively [56]. Higher prevalence of individuals in the pre-contemplation stage among Arabs in Israel compared to minorities in other countries might relate to the lack of tailored policies and interventions to help these individuals move to a more advanced SOC in the readiness to quit smoking. These findings suggest that Israel’s advanced smoking cessation and prevention policies appear to be less effective among Arabs and highlight the importance of exploring factors that can help Arab men move to a more advanced SOC. Determining the characteristics of smokers in different SOC will provide valuable information that can be used to plan and implement tailored smoking cessation interventions.

Previous research has identified different factors that relate to the SOC of the readiness to quit smoking, including demographic and socio-economic characteristics such as age, gender, marital status, SEP, education, and income [31, 57]. In our study, results from the bivariate analysis showed that among the socio-demographic and socio-economic variables, marital status and education level were significantly associated with the SOC. However, these associations became non-significant in the multivariate analysis. Other variables we examined, such as age, primary source of income and employment status, were not significant in the bivariate analysis. Lack of association between SEP and SOC might be explained by the higher prevalence of smoking among Arab men in Israel regardless of education level or SEP [58]. Results from previous studies on the association between demographic and SEP variables and the SOC of the readiness to quit smoking had mixed results, with some finding no association and others finding significant associations [13, 59]. For example, Vicler et al., [60] found that level of education, ethnicity, and race were important for the SOC, while another study that included results of five studies in the US did not find significant associations with demographic or SEP variables [61]. Similar to our results, Choi et al., [62] found that among smokers in Korea, age, education level, and income did not predict the SOC to quit smoking. Gender was the only demographic variable that predicted being at the maintenance stage [62]. Our study did not include women since the prevalence of cigarette smoking among Arab women in Israel is very low (about 5 %) [63]. A study in the Netherlands compared pre-contemplators with contemplators and preparators and found that the level of education, cultural background, and neighborhood conditions correlated with the intention to quit smoking [64]. However, the study compared contemplators and preparators with former smokers [64]. In the current study, we were interested in studying the SOC of the readiness to quit smoking among current smokers; therefore, our sample did not include past smokers. Future studies might want to compare the SOC in current and past smokers among Arab men, and longitudinal research is needed to assess the correlates of quitting behavior.

Although we did not find associations with demographic and socio-economic variables, our findings indicated significant associations between some smoking-related variables and the SOC of the readiness to quit smoking. We found that a longer duration of smoking, higher number of previous attempts to quit, greater knowledge about smoking hazards, and positive attitudes towards smoking prevention and cessation contributed to advanced SOC (contemplation or preparation stages compared to pre-contemplation stage). These results are consistent with findings of a meta-analysis [61], and one cohort study on previous attempts to quit smoking [65]. Nicotine dependence did not contribute to advanced SOC in our study, perhaps due to the fact that we found unexpectedly low dependence levels. It might be that those with low dependence levels are less likely to attempt to quit, a result that was reported in a longitudinal study of low intensity smokers in Mexico [66], and in a study in Egypt [67].

We did not find evidence that social smoking (parents smoking, indoor smoking and number of smokers in a household) contributed to the SOC of the readiness to quit. We assumed that social smoking might be an important factor in the Arab collective society, where cigarettes are distributed during social events such as weddings and funerals. It has previously been suggested that social pressure in Arab societies might contribute to low levels of smoking cessation [68]. However, social norms have been identified as an important factor in quitting smoking [64]. We found that higher social support contributed to a more advanced SOC of the readiness to quit smoking. In addition, our finding that higher levels of anxiety contributed to being at the preparation stage, compared to the pre-contemplation, was unexpected but is important and should be investigated in future research.

Our finding that having a chronic disease was related to a more advanced SOC of the readiness to quit is consistent with findings of two previous cohort studies indicating that, in addition to cigarette costs, health status is the most important reason for quitting smoking [65, 69]. A high proportion of Arab men “wait” until they develop a chronic disease before they are ready to quit smoking, which should set alarms for policy makers and health care providers to intervene before Arab men develop a chronic disease.

In the current study, compared to the pre-contemplation stage, physician advice to quit smoking was associated with the contemplation stage, but not with the preparation stage. The OR of being at the contemplation stage for individuals who received physician advice to quit was almost twice as high as compared to those who did not receive such advice, but physician advice did not significantly increase the OR of being at the preparation stage. This is a very important finding since physician advice was previously found to be cost-effective and increased smoking cessation [70]. However, 60 % of participants in our study reported that their family physician did not advise them to quit smoking. This may be related to a previous finding that prevalence of smoking among physicians is similar to the prevalence of smoking in the general population [3]. Therefore, more effort should be invested by sick funds to help physicians quit smoking. Longitudinal research is needed to assess the long-term effects of physician interventions and their impact on smoking cessation.

The strength of this study is that it is the first study that we know of that examined the SOC of the readiness to quit smoking among Arab men in Israel. The cross-sectional design of the study does not allow for causal inferences of the results, and therefore longitudinal research is recommended. However, the study included, for the first time, a stratified random sample of Arab men who smoke. This enabled us to examine specific factors that relate to the context of these men, who have received less attention in previous research.

## Conclusions

A high proportion of Arab men in Israel who smoke are at the pre-contemplation stage, indicating low readiness to quit smoking. This low readiness might indicate that policies of free-of-charge smoking-cessation technologies in primary health care clinics may be less effective for this minority group. Interventions aimed at increasing smoking cessation among Arab men need to consider the SOC of the readiness to quit and be tailored to fit the needs of Arab men. Generally, statewide smoking cessation programs have been less effective in minority groups. Our study suggests that assessing the SOC might be an important step to designing smoking cessation interventions for ethnic minority groups.
